# Common Glue Dressing

**Published:** 1874-11

**Authors:** 


Therapeutic Use of Common Glue.—A correspondent of the *Scientific American* writes as follows: "A man was running a boring machine with an inch and a quarter auger attached. By some means the sleeve of his shirt caught in the auger, bringing his wrist in contact with the bit, tearing the flesh among the muscles in a frightful manner. He was conducted to my department, (the pattern-shop) and I washed the wound in warm water and glued around it a cloth, which, when dry, shrunk into a rounded shape, holding the wound tight and firm. Once or twice a week, for three or four weeks, I dressed the wound afresh, until it was well. The man never lost an hour's time in consequence. The truth of this statement hundreds can testify to. I use, of course, the best quality of glue."

